# Synchronized and mixed outbreaks of coupled recurrent epidemics

**DOI:** 10.1038/s41598-017-02661-9

**Published:** 2017-05-25

**Authors:** Muhua Zheng, Ming Zhao, Byungjoon Min, Zonghua Liu

**Affiliations:** 1 Department of Physics, East China Normal University, Shanghai, 200062 P. R. China; 2 Levich Institute and Physics Department, City College of New York, New York, New York 10031 USA; 3 College of Physics and Technology, Guangxi Normal University, Guilin, 541004 China

## Abstract

Epidemic spreading has been studied for a long time and most of them are focused on the growing aspect of a single epidemic outbreak. Recently, we extended the study to the case of recurrent epidemics (Sci. Rep. 5, 16010 (2015)) but limited only to a single network. We here report from the real data of coupled regions or cities that the recurrent epidemics in two coupled networks are closely related to each other and can show either synchronized outbreak pattern where outbreaks occur simultaneously in both networks or mixed outbreak pattern where outbreaks occur in one network but do not in another one. To reveal the underlying mechanism, we present a two-layered network model of coupled recurrent epidemics to reproduce the synchronized and mixed outbreak patterns. We show that the synchronized outbreak pattern is preferred to be triggered in two coupled networks with the same average degree while the mixed outbreak pattern is likely to show for the case with different average degrees. Further, we show that the coupling between the two layers tends to suppress the mixed outbreak pattern but enhance the synchronized outbreak pattern. A theoretical analysis based on microscopic Markov-chain approach is presented to explain the numerical results. This finding opens a new window for studying the recurrent epidemics in multi-layered networks.

## Introduction

Epidemic spreading in complex networks has been well studied and a lot of great progress have been achieved such as the infinitesimal threshold^1–6^, reaction-diffusion model^7–10^, flow driven epidemic^11–15^, and objective spreading etc.^16, 17^, see the review refs 18–20. for details. Recently, the attention has been moved to the case of multilayer networks^18, 21–31^, which represent the interactions between different real-world networks such as critical infrastructure^32–34^, transportation networks^35, 36^, living organisms^37–39^, and social networks^21, 40^ etc. These models enable us to determine how the interplay between network structures influences the dynamic processes taking place on them^41–50^. For instance, a pathogen spreads on a human contact network abetted by global and regional transportation networks^21^. Due to their ubiquitous applications in complex systems^51–55^, the understanding of the properties and dynamic processes in multilayered networks carries great practical significance.

Two of the most successful models used to describe epidemic spreading are the susceptible-infected-susceptible (SIS) and susceptible-infected-refractory (SIR) models. Mark *et al*. used the SIR model to multilayered networks in 2012^41^. Very interesting, they found a mixed phase in weakly coupled networks where an epidemic occurs in one network but does not spread to the coupled network. Saumell-Mendiola *et al*. used the SIS model to multilayered networks also in 2012^42^. However, they found that such a mixing phase doesn’t exist in both analytic and simulation results. In their work, they mainly focused on the epidemic threshold and studied how epidemics spread from one network to another.

**Figure 1:**
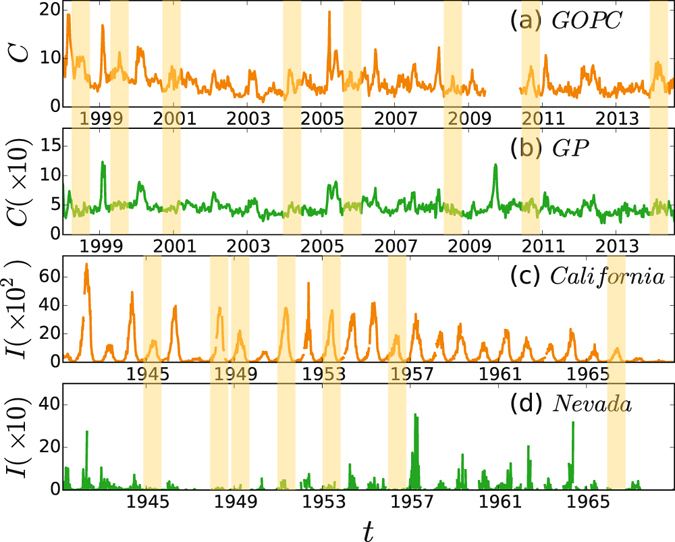
Time series of recurrent epidemics in two coupled regions or cities. (**a**) and (**b**) represent the weekly consultation rates of influenza-like illness (per 1000 consultations) from 1998 to 2014 in Hong Kong for the General Out-Patient Clinics (GOPC) and the General Practitioners (GP), respectively, where the data from 2009/6/13 to 2010/5/23 in (**a**) are not available and the value of *C* in (**b**) is from 0 to 150. (**c**) and (**d**) represent the time series of reported weekly measles infective cases *I* in California and Nevada, respectively.

All these studies are focused on the growing aspect of a single epidemic outbreak, no matter it is one layer or multilayered networks. However, in realistic situations, the empirical data shows that epidemic is recurrent, i.e. with outbreaks from time to time^56–60^. Thus, we recently extended the study to the case of recurrent epidemics^57^, but limited only to a single network. Considering that the interactions between different networks are ubiquitous, we here recheck several real data of coupled regions or cities such as the General Out-Patient Clinics (GOPC) network and its coupled General Practitioners (GP) network of Hong Kong (see Fig. 1(a) and (b)), the coupled regional networks of California and Nevada (see Fig. 1(c) and (d)), the coupled regional networks of Arizona and California (see Fig. a(a) and (b) in SI), the coupled city networks of Boston and Fall River (see Fig. 1(c) and (d) in SI), and the coupled city networks of Los Angeles and Sacramento (see Fig. 1(e) and (f) in SI). We interestingly find that their recurrent epidemics are closely related to each other. Moreover, we find that the coupled time series of recurrent epidemics can show either synchronized outbreak pattern where outbreaks occur simultaneously in both networks or mixed outbreak pattern where outbreaks occur only in one network but do not in another one. This finding calls our great interest and motivates us to study its underlying mechanism. In this sense, we believe that it is very necessary to further extend the study of recurrent epidemics to the case of multilayered networks.

In this work, we present a two-layered network model of coupled recurrent epidemics to reproduce the synchronized and mixed outbreak patterns. To guarantee the appearance of recurrent outbreaks, we choose the susceptible-infected-refractory-susceptible (SIRS) model for each node of network and let the infectious rate be time dependent, symbolizing the larger annual variation of environment. By this model, we find that the average degrees of both the intra- and inter-networks play key roles on the emergence of synchronized and mixed outbreak patterns. The synchronized outbreak pattern tends to be triggered in two coupled networks with the same average degree while the mixed outbreak pattern is likely to show for the case with different average degrees. Further, we show that the increasing of coupling strength, i.e. either the inter-layer infection rate or inter-layer average degree, will tend to suppress the mixed outbreak pattern but enhance the synchronized outbreak pattern. A theoretical analysis based on microscopic Markov-chain approach is presented to explain the numerical results. This finding may be of significance to the long-term prediction and control of recurrent epidemics in multi-areas or cities.

## Results

### The synchronized and mixed outbreak patterns of recurrent epidemics in real data

Monitoring epidemic spreading is vital for us to prevent and control infectious diseases. For this purpose, Hong Kong Department of Health launched a sentinel surveillance system to collect data of infectious diseases, aiming to analyze and predict the trend of infectious spreading in different regions of Hong Kong. In this system, there are about 64 General Out-Patient Clinics (GOPC) and 50 General Practitioners (GP), which form two sentinel surveillance networks of Hong Kong^56^, respectively. By these two networks, we can obtain the weekly consultation rates of influenza-like illness (per 1000 consultations). Figure 1(a) and (b) show the collected data from 1998 to 2014 for the GOPC and GP, respectively, where the data from 2009/6/13 to 2010/5/23 in (a) was not collected by Hong Kong Department of Health and the value of *C* in (b) is from 0 to 150. This weekly consultation rates of influenza-like illness can well reflect the overall influenza-like illness activity in Hong Kong. From the data in Fig. 1(a) and (b) we easily find that there are intermittent peaks, marking the recurrent outbreaks of epidemics. By a second check on the data in Fig. 1(a) and (b) we interestingly find that some peaks occur simultaneously in the two networks at the same time, indicating the appearance of synchronized outbreak pattern. While other peaks appear only in Fig. 1(a) but not in Fig. 1(b), indicating the existence of mixed outbreak pattern (see the light yellow shaded areas).

Is this finding of synchronized and mixed outbreak patterns in recurrent epidemics a specific phenomenon only in Hong Kong? To figure out its generality, we have checked a large number of other recurrent infectious data in different pathogens and in different states and cities in the United States and found the similar phenomenon. Figure 1(c) and (d) show the data of weekly measles infective cases *I* in the states of California and Nevada, respectively, which were obtained from the USA National Notifiable Diseases Surveillance System as digitized by Project Tycho^58–60^. As California is adjacent to Nevada in west coast of the United States, their climatic conditions are similar. Thus, they can be also considered as two coupled networks. Three more these kinds of examples have been shown in Fig. 1 of SI where the coupled networks are based on states-level influenza data and cities-level measles data, respectively. Therefore, the synchronized and mixed outbreak patterns are general in recurrent epidemics. We will explain their underlying mechanisms in the next subsection.

### A two-layered network model of coupled recurrent epidemics

Two classical models of epidemic spreading are the Susceptible-Infected-Susceptible (SIS) model and Susceptible-Infected-Refractory (SIR) model^18^. In the SIS model, a susceptible node will be infected by an infected neighbor with rate *β*. In the meantime, each infected node will recover with a probability *γ* at each time step. After the transient process, the system reaches a stationary state with a constant infected density *ρ*_*I*_. Similarly, in the SIR model, each node can be in one of the three states: Susceptible, Infected, and Refractory. At each time step, a susceptible node will be infected by an infected neighbor with rate *β* and an infected node will become refractory with probability *γ*. The infection process will be over when there is no infected *I*. These two models have been widely used in a variety of situations. However, it was pointed out that both the SIS and SIR models are failed to explain the recurrence of epidemics in real data^57, 61^.

**Figure 2:**
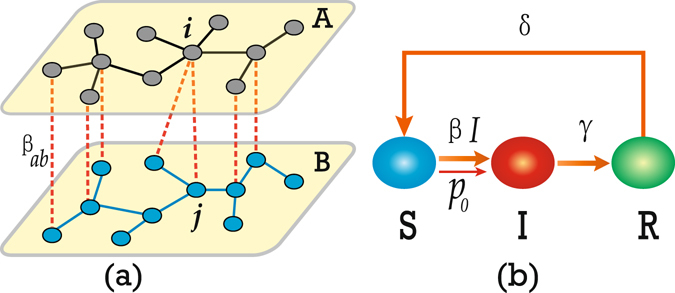
Schematic figure of the epidemic model to reproduce the synchronized and mixed outbreak patterns. (**a**) Schematic figure of the two-layered network, where the “black”, “blue” and “red” lines represent the links of the networks ${\mathscr{A}}, {\mathcal B}$ and the inter-network ${\mathscr{A}} {\mathcal B}$, respectively. *β*_*ab*_ denotes the infectious rate through one interconnection between ${\mathscr{A}}\,{\rm{and}}\, {\mathcal B}$. (b) Schematic figure of the extended SIRS model for each node in ${\mathscr{A}}\,{\rm{and}}\, {\mathcal B}$, where the symbols *S*, *I* and *R* represent the susceptible, infectious, and refractory states, respectively, and the parameters *β*, *γ* and *δ* represent the infectious, refractory and recovery rates, respectively. *p*_0_ denotes the probability for a susceptible node to be naturally infected by environment or other factors.

To reproduce the synchronized and mixed outbreak patterns, we here present a two-layered network model of coupled recurrent epidemics, shown in Fig. 2 where (a) represents its schematic figure of network topology and (b) denotes the epidemic model at each node. In Fig. 2(a), two networks ${\mathscr{A}}\,{\rm{and}}\, {\mathcal B}$ are coupled through some interconnections between them, which form the inter-network ${\mathscr{A}} {\mathcal B}$. For the sake of simplicity, we let the two networks ${\mathscr{A}}\,{\rm{and}}\, {\mathcal B}$ have the same size *N*_*a*_ = *N*_*b*_. We let 〈*k*_*a*_〉, 〈*k*_*b*_〉, and 〈*k*_*ab*_〉 represent the average degrees of the networks ${\mathscr{A}}, {\mathcal B} \,{\rm{and}}\,{\mathscr{A}} {\mathcal B}$, respectively, see *Methods* for details. In Fig. 2(b), the epidemic model is adopted from our previous work^57^ by two steps. In step one, we extend the SIR model to a Susceptible-Infected-Refractory-Susceptible (SIRS) model where a refractory node will become susceptible again with probability *δ*. In step two, we let each susceptible node have a small probability *p*_0_ to be infected, which represents the natural infection from environment. Moreover, we let the infectious rate *β*(*t*) be time dependent, representing its annual and seasonal variations etc. To distinguish the function of the interconnections from that of those links in ${\mathscr{A}}\,{\rm{and}}\, {\mathcal B}$, we let *β*_*ab*_ be the inter-layer infectious rate. In this way, the interaction between ${\mathscr{A}}\,{\rm{and}}\, {\mathcal B}$ can be described by the tunable parameter *β*_*ab*_.

**Figure 3:**
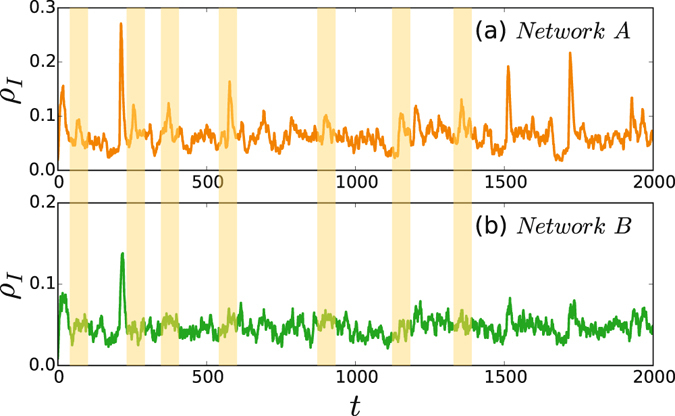
Reproduced time series of recurrent epidemics in the two-layered network model. (**a**) and (**b**) represent the evolutions of the infected density *ρ*_*I*_ in the two-layered networks ${\mathscr{A}}\,{\rm{and}}\, {\mathcal B}$, respectively, where the parameters are taken as 〈*k*_*a*_〉 = 6.5, 〈*k*_*b*_〉 = 1.5, 〈*k*_*ab*_〉 = 1.0, $\beta (t)\sim {\mathscr{N}}(0.1,{0.1}^{2})$, *β*_*ab*_ = 0.09, *γ* = 0.2, *δ* = 0.02, *p*_0_ = 0.01, and *N*_*a*_ = *N*_*b*_ = 1000.

In numerical simulations, we let both the networks ${\mathscr{A}}\,{\rm{and}}\, {\mathcal B}$ be the Erdös-Rényi (ER) random networks^62^. To guarantee a recurrent epidemics in each of ${\mathscr{A}}\,{\rm{and}}\, {\mathcal B}$, we follow ref. 57 to let *β*(*t*) be the truncated Gaussian distribution ${\mathscr{N}}(0.1,{0.1}^{2})$ and choose *p*_0_ = 0.01. Figure 3(a) and (b) show the evolutions of the infected density *ρ*_*I*_ in ${\mathscr{A}}\,{\rm{and}}\, {\mathcal B}$, respectively, where the parameters are taken as 〈*k*_*a*_〉 = 6.5, 〈*k*_*a*_〉 = 1.5, 〈*k*_*ab*_〉 = 1.0, and *β*_*ab*_ = 0.09. It is easy to observe that some peaks of *ρ*_*I*_ appear simultaneously in ${\mathscr{A}}\,{\rm{and}}\, {\mathcal B}$, indicating the synchronized outbreak pattern. We also notice that some peaks of *ρ*_*I*_ in ${\mathscr{A}}$ do not have corresponding peaks in ${\mathcal B}$ (see the light yellow shadowed areas in Fig. 3(a) and (b)), indicating the mixed outbreak pattern. Moreover, we do not find the contrary case where there are peaks of *ρ*_*I*_ in ${\mathcal B}$ but no corresponding peaks in ${\mathscr{A}}$, which is also consistent with the empirical observations in Fig. 1(a–d).

### Mechanism of the synchronized and mixed outbreak patterns

To understand the phenomenon of synchronized and mixed outbreak patterns better, we here study their underlying mechanisms. A key quantity for the phenomenon is the outbreak of epidemic, i.e. the peaks in the time series of Fig. 1. Notice that a peak is usually much higher than its background oscillations. To pick out a peak, we need to define its background/baseline first. As the distributions of both the real data in Fig. 1 and numerical simulations in Fig. 3 are approximately satisfied the normal distribution (see Fig. 2 SI), we define the baseline as *μ* + 3*σ* with *μ* and *σ* being the mean and standard deviation, respectively, which contains about 99.7% data in the normal distribution^63^. Then, we can count the number of outbreaks in a measured time *t*. Let *n* be the average number of outbreaks in realizations of the same evolution *t*. Larger *n* implies more frequent outbreaks. Let ${\rm{\Delta }}n=|n({\mathscr{A}})-n( {\mathcal B} )|$ be the difference of outbreak numbers between the networks ${\mathscr{A}}\,{\rm{and}}\, {\mathcal B}$. Larger Δ*n* implies more frequent emergence of the mixed outbreak pattern. In particular, the mixed outbreak pattern will disappear when Δ*n* = 0.

**Figure 4:**
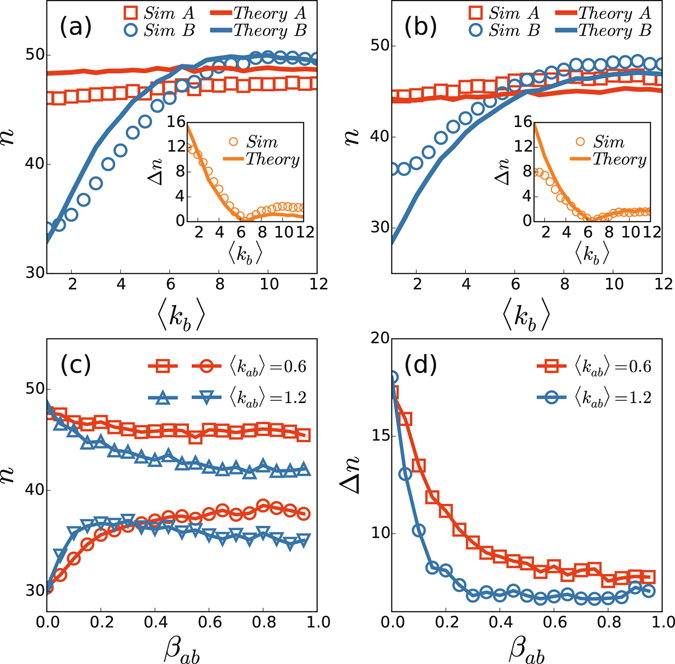
(**a**) and (**b**) Dependence of *n* on the average degree 〈*k*_*b*_〉 of network ${\mathcal B}$ for *β*_*ab*_ = 0.09 and 0.3, respectively, where the average degree of network ${\mathscr{A}}$ is fixed as 〈*k*_*a*_〉 = 6.5, 〈*k*_*ab*_〉 = 1.0, and the insets show the dependence of ${\rm{\Delta }}n=|n({\mathscr{A}})-n( {\mathcal B} )|$ on 〈*k*_*b*_〉. The solid lines represent the theoretical results from Eqs (6) and (7). (**c**) Dependence of *n* on *β*_*ab*_ with 〈*k*_*a*_〉 = 6.5 and 〈*k*_*b*_〉 = 1.5 where the “squares” and “circles” represent the case of 〈*k*_*ab*_〉 = 0.6 for the networks ${\mathscr{A}}\,{\rm{and}}\, {\mathcal B}$, respectively, and the “up triangles” and “down triangles” represent the case of 〈*k*_*ab*_〉 = 1.2 for the networks ${\mathscr{A}}\,{\rm{and}}\, {\mathcal B}$, respectively. (**d**) Dependence of Δ*n* on *β*_*ab*_ with 〈*k*_*a*_〉 = 6.5 and 〈*k*_*b*_〉 = 1.5 where the “squares” and “circles” represent the cases of 〈*k*_*ab*_〉 = 0.6 and 1.2, respectively. The other parameters are set as $\beta (t)\sim {\mathscr{N}}(0.1,{0.1}^{2})$, *γ* = 0.2, *δ* = 0.02, *p*_0_ = 0.01, and *N*_*a*_ = *N*_*b*_ = 1000. All the results are averaged over 1000 independent realizations with the simulation steps *t* = 20000.

We are interested in how the average degrees and coupling influence the numbers *n* and Δ*n*. Figure 4(a) and (b) show the dependence of *n* on the average degree 〈*k*_*b*_〉 of network ${\mathcal B}$ for *β*_*ab*_ = 0.09 and 0.3, respectively, where the average degree of network ${\mathscr{A}}$ is fixed as 〈*k*_*a*_〉 = 6.5. It is easy to see from Fig. 4(a) and (b) that the number $n( {\mathcal B} )$ of network ${\mathcal B}$ will gradually increase with the increase of 〈*k*_*b*_〉, while the number $n({\mathscr{A}})$ of network ${\mathscr{A}}$ keeps approximately constant, indicating that a larger 〈*k*_*b*_〉 is in favor of the recurrent outbreaks. Specifically, $n( {\mathcal B} )$ will reach $n({\mathscr{A}})$ when 〈*k*_*b*_〉 is increased to the value of 〈*k*_*b*_〉 = 〈*k*_*a*_〉 = 6.5, see the insets in Fig. 4(a) and (b) for the minimum of Δ*n*. For details, Fig. 3 in SI shows the evolution of infected densities *ρ*_*I*_ for the cases of 〈*k*_*a*_〉 = 〈*k*_*b*_〉, confirming the result of Δ*n* = 0 in Fig. 4(a) and (b). On the other hand, comparing the two insets in Fig. 4 (a) and (b), we find that Δ*n* in Fig. 4(a) is greater than the corresponding one in Fig. 4(b), indicating that a larger *β*_*ab*_ is in favor of suppressing the mixed outbreak pattern. These results can be also theoretically explained by the microscopic Markov-chain approach, see *Methods* for details. The solid lines in Fig. 4 (a) and (b) represent the theoretical results from Eqs (6) and (7). It is easy to see that the theoretical results are consistent with the numerical simulations very well.

Figure 4(c) and (d) show the influences of the coupling parameters such as *β*_*ab*_ and *k*_*ab*_ for the outbreak number *n* and the difference of outbreak number Δ*n*, respectively, where 〈*k*_*a*_〉 = 6.5 and 〈*k*_*b*_〉 = 1.5. From Fig. 4(c) we see that for the case of *k*_*ab*_ = 0.6, $n({\mathscr{A}})$ is an approximate constant and $n( {\mathcal B} )$ gradually increase with *β*_*ab*_. While for the case of ${k}_{ab}\mathrm{=1.2}$, both $n({\mathscr{A}})$ and $n( {\mathcal B} )$ change with *β*_*ab*_, indicating that both ${k}_{ab}$ and *β*_*ab*_ take important roles in the synchronized and mixed outbreak patterns. From Fig. 4(d) we see that the case of *k*_*ab*_ = 1.2 decreases faster than the case of *k*_*ab*_ = 0.6, indicating that both the larger *k*_*ab*_ and larger *β*_*ab*_ are in favor of the synchronized outbreak pattern. That is, the stronger coupling will suppress the mixed outbreak pattern but enhance the synchronized outbreak pattern. On the contrary, the weaker coupling is in favor of the mixed epidemic outbreak pattern but suppress the synchronized outbreak pattern. For details, Fig. 4 in SI shows the evolution of infected densities *ρ*_*I*_ for different *β*_*ab*_, confirming the above results.

### Coupling induced correlation between the epidemics of the two networks

**Figure 5:**
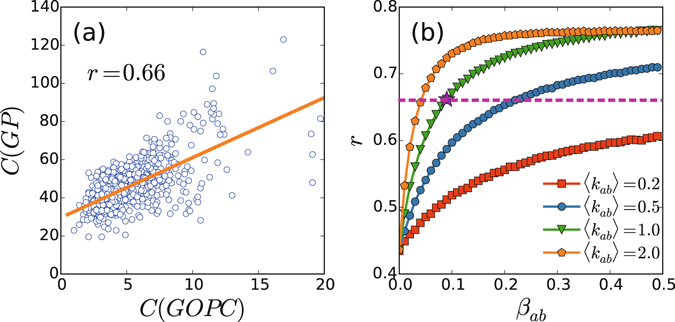
Correlation between the two coupled layers. (**a**) Correlation between the two time series of GOPC and GP. (**b**) Dependence of the correlation coefficient *r* on *β*_*ab*_ for 〈*k*_*ab*_〉 = 0.2, 0.5, 1.0 and 2.0, respectively, where the purple “star” and its related dashed line represent the point of *r* = 0.66. Other parameters are the same as in Fig. 3(a) and (b).

The coupling between the two layers is represented by the pair of variables (*β*_*ab*_, 〈*k*_*ab*_〉). Qualitatively, larger *β*_*ab*_ and 〈*k*_*ab*_〉 represent stronger coupling. To quantitatively represent the effects of *β*_*ab*_ and 〈*k*_*ab*_〉, we here measure the cross-correlation, *r*, defined in Eq. (19), which can show some information beyond the synchronized and mixed outbreak patterns. By Eq. (19) we first calculate the coefficient *r* between the two time series of GOPC and GP and find *r* = 0.66, indicating that these two data are highly correlated. Figure 5(a) shows the correspondence between the two time series of GOPC and GP. Then, we check the influence of coupling on the coefficient *r*. Figure 5(b) shows the dependence of *r* on *β*_*ab*_ for 〈*k*_*ab*_〉 = 0.2, 0.5, 1.0 and 2.0, respectively. We see that *r* increases with *β*_*ab*_ for a fixed 〈*k*_*ab*_〉 and also increases with 〈*k*_*ab*_〉 for a fixed *β*_*ab*_, indicating the enhanced correlation by the coupling strength. Very interesting, we find that for the case of 〈*k*_*ab*_〉 = 1.0 in Fig. 5(b), the point of *r* = 0.66 corresponds to 〈*β*_*ab*_〉 = 0.09 (see the purple “star”), implying that the coupling in Fig. 5(a) is equivalent to the case of 〈*k*_*ab*_〉 = 1.0 and 〈*β*_*ab*_〉 = 0.09 in Fig. 5(b). In this sense, we may draw a horizontal line passing the purple “star” in Fig. 5(b) (see the dashed line) and its crossing points with all the curves there will also represent the equivalent coupling in Fig. 5(a). However, we notice that the horizontal line has no crossing point with the curve of 〈*k*_*ab*_〉 = 0.2, indicating that there is a threshold of 〈*k*_*ab*_〉 for the appearance of *r* = 0.66 in Fig. 5(a).

It is maybe helpful to understand the influence of (*β*_*ab*_, 〈*k*_*ab*_〉) on epidemics from the aspect of purely coupling in network structure. We know that a critical problem in coupled networks is when they behave as separate networks vs when they behave as a solid single network^64, 65^. In our case here, the synchrony of epidemic in the two layers corresponds the case of strong coupling while the mixed pattern represents the case of weak coupling. Therefore, the synchrony and mixed patterns also reflect the influence of network structure on its dynamics. On the other hand, we notice from Fig. 5(b) that there is a finite value of *r* ≈ 0.43 when *β*_*ab*_ = 0, implying that the same infection probability *β*(*t*) in the two networks do have a contribution to the correlation *r*. However, the further increase of *r* in Fig. 5(b) reflects the influence from the coupling parameters of (*β*_*ab*_, 〈*k*_*ab*_〉). From Fig. 5(b) we see that for a fixed *β*_*ab*_, *r* increases with 〈*k*_*ab*_〉, indicating the influence of 〈*k*_*ab*_〉. While for a fixed 〈*k*_*ab*_〉, *r* increases with *β*_*ab*_, indicating the influence of *β*_*ab*_. Therefore, the further increase of correlation from *r* ≈ 0.43 do come from the coupling between the two time series.

## Discussion

The dependence of the synchronized and mixed outbreak patterns on the average degrees of networks ${\mathscr{A}}\,{\rm{and}}\, {\mathcal B}$ may be also understood from the aspect of their epidemic thresholds. By the theoretical analysis in *Methods* we obtain the epidemic thresholds as ${\beta }_{c}^{A}=\frac{\gamma }{\langle {k}_{a}\rangle }\,{\rm{and}}\,{\beta }_{c}^{B}=\frac{\gamma }{\langle {k}_{b}\rangle }$ in Eq. (18), when the two networks are weakly coupled. For the case of 〈*k*_*a*_〉 > 〈*k*_*b*_〉, we have ${\beta }_{c}^{A} < {\beta }_{c}^{B}$. When *β* satisfies $\beta  < {\beta }_{c}^{A} < {\beta }_{c}^{B}$, the epidemics cannot survive in each of networks ${\mathscr{A}}\,{\rm{and}}\, {\mathcal B}$. Thus, the infected fraction will be approximately zero, i.e. no epidemic outbreak in both ${\mathscr{A}}$ and ${\mathcal B}$. It should be noticed that this result just holds for the case of weakly coupling. When coupling is strong, it is possible for epidemic to occur in the coupled network even when *β* is below the epidemic threshold of each layer^24^. When *β* satisfies $\beta  > {\beta }_{c}^{B} > {\beta }_{c}^{A}$, the epidemics will survive in both networks ${\mathscr{A}}\,{\rm{and}}\, {\mathcal B}$, indicating an outbreak will definitely occur in both of them. These two cases are trivial. However, when *β* is in between ${\beta }_{c}^{A}\,{\rm{and}}\,{\beta }_{c}^{B}$, some interesting results may be induced by coupling. When coupling is weak, it is possible for epidemic to outbreak only in network ${\mathscr{A}}$ but not in network ${\mathcal B}$. When coupling is increased slightly, it will be also possible for epidemic to outbreak sometimes in network ${\mathcal B}$, i.e. resulting a mixed outbreak pattern. Once coupling is further increased to large enough, a synchronized outbreak pattern will be resulted.

So far, the reported results are from the ER random networks ${\mathscr{A}}\,{\rm{and}}\, {\mathcal B}$. We are wondering whether it is possible to still observe the phenomenon of the synchronized and mixed outbreak patterns in other network topologies. For this purpose, we here take the scale-free network^62^ as an example. Very interesting, by repeating the above simulation process in scale-free networks we have found the similar phenomenon as in ER random networks, see Figs 5–7 in SI for details. Therefore, the synchronized and mixed outbreak patterns are a general phenomena in multi-layered epidemic networks.

In sum, the epidemic spreading has been well studied in the past decades, mainly focused on both the single and multi-layered networks. However, only a few works focused on the aspect of recurrent epidemics, including both the models in homogeneous population^61^ and our recent model in a single network^57^. We here report from the real data that the epidemics from different networks are in fact not isolated but correlated, implying that they should be considered as a multi-layered network. Motivated by this discovery, we present a two-layered network model to reproduce the correlated recurrent epidemics in coupled networks. More importantly, we find that this model can reproduce both the synchronized and mixed outbreak patterns in real data. The two-layered network favors to show the synchronized pattern when the average degrees of the two coupled networks have a large difference and shows the mixed pattern when their average degrees are very close. Besides the degree difference between the two networks, the coupling strength between the two layers has also significant influence to the synchronized and mixed outbreak patterns. We show that both the larger *β*_*ab*_ and larger 〈*k*_*ab*_〉 are in favor of the synchronized pattern but suppress the mixed pattern. This finding thus shows a new way to understand the epidemics in realistic multi-layered networks. Its further studies and potential applications in controlling the recurrent epidemics may be an interesting topic in near future.

## Methods

### A two-layered network model of recurrent epidemic spreading

We consider a two-layered network model with coupling between its two layers, i.e. the networks ${\mathscr{A}}\,{\rm{and}}\, {\mathcal B}$ in Fig. 2(a). We let the two networks have the same size *N*_*a*_ = *N*_*a*_ = *N* and their degree distributions *P*_*A*_(*k*) and *P*_*B*_(*k*) be different. We may imagine the network ${\mathscr{A}}$ as a human contact network for one geographic region and the network ${\mathcal B}$ for a separated region. Each node in the two-layered network has two kinds of links, i.e. intra-links within ${\mathscr{A}}$ or ${\mathcal B}$ and the interconnection between ${\mathscr{A}}\,{\rm{and}}\, {\mathcal B}$. The former consists of the degree distributions *P*_*A*_(*k*) and *P*_*B*_(*k*) while the latter the interconnection network. We let 〈*k*_*a*_〉, 〈*k*_*b*_〉, and 〈*k*_*ab*_〉 represent the average degrees of networks ${\mathscr{A}}, {\mathcal B}$ and interconnection network ${\mathscr{A}} {\mathcal B}$, respectively. In details, we firstly generate two separated networks ${\mathscr{A}}\,{\rm{and}}\, {\mathcal B}$ with the same size *N* and different degree distributions *P*_*A*_(*k*) and *P*_*B*_(*k*), respectively. Then, we add links between ${\mathscr{A}}\,{\rm{and}}\, {\mathcal B}$. That is, we randomly choose two nodes from ${\mathscr{A}}\,{\rm{and}}\, {\mathcal B}$ and then connect them if they are not connected yet. Repeat this process until the steps we planned. In this way, we obtain an uncorrelated two-layered network.

To discuss epidemic spreading in the two-layered network, we use the extended SIRS model, see Fig. 2(b) for its schematic illustration. In this model, a susceptible node has three ways to be infected. The first one is the natural infection from environment or unknown reasons, represented by a small probability *p*_0_. The second one is the infection from contacting with infected individuals in the network ${\mathscr{A}}$ (or ${\mathcal B}$), represented by *β*(*t*). And the third one is the infection from another network, represented by *β*_*ab*_ (see Fig. 2(a)). Thus, a susceptible node will become infected with a probability $1-(1-{p}_{0}){(1-\beta (t))}^{{k}^{inf}}{(1-{\beta }_{ab})}^{{k}_{ab}^{inf}}$ where *k* ^*inf*^ is the infected neighbors in the same network and ${k}_{ab}^{inf}$ is the infected neighbors in another network. At the same time, an infected node will become refractory by a probability *γ* and a refractory node will become susceptible again by a probability *δ*.

In numerical simulations, the dependence of *β*(*t*) on time is implemented as follows^57^: we divide the total time *t* into multiple segments with length *T* and let *T* = 52, corresponding to the 52 weeks in one year. We let *β*(*t*) be a constant in each segment, which is randomly chosen from the truncated Gaussian distribution ${\mathscr{N}}(0.1,{0.1}^{2})$. Once a *β*(*t*) < 0 or *β*(*t*) > 1 is chosen, we discard it and then choose a new one. At the same time, we fix *γ* = 0.2 and *δ* = 0.2 and set *β*_*ab*_ as the tunable parameter.

### A theoretical analysis based on microscopic Markov-chain approach

Let ${P}_{i,A}^{S}(t),{P}_{i,A}^{I}(t),{P}_{i,A}^{R}(t)$ be the probabilities for node *i* in network ${\mathscr{A}}$ to be in one of the three states of *S*, *I* and *R* at time *t*, respectively. Similarly, we have ${P}_{i,B}^{S}(t),{P}_{i,B}^{I}(t)\,{\rm{and}}\,{P}_{i,B}^{R}(t)$ in network ${\mathcal B}$. They satisfy the conservation law

1 $${P}_{i,A}^{S}(t)+{P}_{i,A}^{I}(t)+{P}_{i,A}^{R}(t)=1,\,{P}_{i,B}^{S}(t)+{P}_{i,B}^{I}(t)+{P}_{i,B}^{R}(t)=1.$$

According to the Markov-chain approach^43, 57, 66–68^, we introduce

$${\rho }_{S}^{A}(t)=\frac{1}{N}\sum _{i=1}^{N}{P}_{i,A}^{S}(t),\,{\rho }_{I}^{A}(t)=\frac{1}{N}\sum _{i=1}^{N}{P}_{i,A}^{I}(t),\,{\rho }_{R}^{A}(t)=\frac{1}{N}\sum _{i=1}^{N}{P}_{i,A}^{R}(t),$$

$${\rho }_{S}^{B}(t)=\frac{1}{N}\sum _{i=1}^{N}{P}_{i,B}^{S}(t),\,{\rho }_{I}^{B}(t)=\frac{1}{N}\sum _{i=1}^{N}{P}_{i,B}^{I}(t),\,{\rho }_{R}^{B}(t)=\frac{1}{N}\sum _{i=1}^{N}{P}_{i,B}^{R}(t),$$

where ${\rho }_{S}^{A}(t),{\rho }_{I}^{A}(t)\,{\rm{and}}\,{\rho }_{R}^{A}(t)$ represent the densities of susceptible, infected, and refractory individuals at time *t* in network ${\mathscr{A}}$, respectively. Similarly, we have ${\rho }_{S}^{B}(t),{\rho }_{I}^{B}(t)\,{\rm{and}}\,{\rho }_{R}^{B}(t)$ in network ${\mathcal B}$.

Let, ${q}_{i,A}^{I,R}(t),{q}_{i,A}^{S,I}(t)\,{\rm{and}}\,{q}_{i,A}^{R,S}(t)$ be the transition probabilities from the state *S* to *I*, *I* to *R*, and *R* to *S* in network ${\mathscr{A}}$, respectively. By the Markov chain approach^66, 68^ we have

2 $$\begin{matrix}{q}_{i,A}^{S,I}(t) & = & 1-(1-{p}_{0})\prod _{l\in {\Lambda }_{i,A}}(1-\beta (t){P}_{l,A}^{I}(t))\prod _{v\in {\Lambda }_{i,B}}(1-{\beta }_{ab}{P}_{v,B}^{I}(t)),\\ {q}_{i,A}^{I,R}(t) & = & \gamma ,\\ {q}_{i,A}^{R,S}(t) & = & \delta ,\end{matrix}$$

where Λ_*i*, *A*_ represents the neighboring set of node *i* in network ${\mathscr{A}}$. The term (1 − *p*_0_) in Eq. (2) represents the probability that node *i* is not infected by the environment. The term ${\prod }_{l\in {\Lambda }_{i,A}}(1-\beta (t){P}_{l,A}^{I}(t))$ is the probability that node *i* is not infected by the infected neighbors in network ${\mathscr{A}}$. While the term ${\prod }_{v\in {\Lambda }_{i,B}}(1-{\beta }_{ab}{P}_{v,B}^{I}(t))$ is the probability that node *i* is not infected by the infected neighbors in another network. Thus, $(1-{p}_{0}){\prod }_{l\in {\Lambda }_{i,A}}(1-\beta (t){P}_{l,A}^{I}(t)){\prod }_{v\in {\Lambda }_{i,B}}(1-{\beta }_{ab}{P}_{v,B}^{I}(t))$ is the probability for node *i* to be in the susceptible state. Similarly, for the node in network ${\mathcal B}$, we have

3 $$\begin{matrix}{q}_{i,B}^{S,I}(t) & = & 1-(1-{p}_{0})\prod _{l\in {\Lambda }_{i,B}}(1-\beta (t){P}_{l,B}^{I}(t))\prod _{v\in {\Lambda }_{i,A}}(1-{\beta }_{ab}{P}_{v,A}^{I}(t)),\\ {q}_{i,B}^{I,R}(t) & = & \gamma ,\\ {q}_{i,B}^{R,S}(t) & = & \delta .\end{matrix}$$

Based on these analysis, we formulate the following difference equations

4 $$\begin{matrix}{P}_{i,A}^{S}(t+1) & = & {P}_{i,A}^{S}(t)(1-{q}_{i,A}^{S,I}(t))+{P}_{i,A}^{R}(t){q}_{i,A}^{R,S}(t),\\ {P}_{i,A}^{I}(t+1) & = & {P}_{i,A}^{I}(t)(1-{q}_{i,A}^{I,R}(t))+{P}_{i,A}^{S}(t){q}_{i,A}^{S,I}(t),\\ {P}_{i,A}^{R}(t+1) & = & {P}_{i,A}^{R}(t)(1-{q}_{i,A}^{R,S}(t))+{P}_{i,A}^{I}(t){q}_{i,A}^{I,R}(t).\end{matrix}$$

5 $$\begin{matrix}{P}_{i,B}^{S}(t+1) & = & {P}_{i,B}^{S}(t)(1-{q}_{i,B}^{S,I}(t))+{P}_{i,B}^{R}(t){q}_{i,B}^{R,S}(t),\\ {P}_{i,B}^{I}(t+1) & = & {P}_{i,B}^{I}(t)(1-{q}_{i,B}^{I,R}(t))+{P}_{i,B}^{S}(t){q}_{i,B}^{S,I}(t),\\ {P}_{i,B}^{R}(t+1) & = & {P}_{i,B}^{R}(t)(1-{q}_{i,B}^{R,S}(t))+{P}_{i,B}^{I}(t){q}_{i,B}^{I,R}(t).\end{matrix}$$

The first term on the right-hand side of the first equation of Eq. (4) is the probability that node *i* is remained as susceptible state. The second term stands for the probability that node *i* is changed from refractory to susceptible state. Similarly, we have the same explanation for the other equations of Eqs (4) and (5). Substituting Eqs (2) and (3) into Eqs (4) and (5), we obtain the microscopic Markov dynamics as follows

6 $$\begin{matrix}{P}_{i,A}^{S}(t+1) & = & {P}_{i,A}^{S}(t)[(1-{p}_{0})\prod _{l\in {\Lambda }_{i,A}}(1-\beta (t){P}_{l,A}^{I}(t))\prod _{v\in {\Lambda }_{i,B}}(1-{\beta }_{ab}{P}_{v,B}^{I}(t))]+{P}_{i,A}^{R}(t)\delta ,\\ {P}_{i,A}^{I}(t+1) & = & {P}_{i,A}^{I}(t)(1-\gamma )+{P}_{i,A}^{S}(t)\times [1-(1-{p}_{0})\prod _{l\in {\Lambda }_{i,A}}(1-\beta (t){P}_{l,A}^{I}(t))\prod _{v\in {\Lambda }_{i,B}}(1-{\beta }_{ab}{P}_{v,B}^{I}(t))],\\ {P}_{i,A}^{R}(t+\mathrm{1)} & = & {P}_{i,A}^{R}(t)(1-\delta )+{P}_{i,A}^{I}(t)\gamma .\end{matrix}$$

7 $$\begin{matrix}{P}_{i,B}^{S}(t+1) & = & {P}_{i,B}^{S}(t)[(1-{p}_{0})\prod _{l\in {\Lambda }_{i,B}}(1-\beta (t){P}_{l,B}^{I}(t))\prod _{v\in {\Lambda }_{i,A}}(1-{\beta }_{ab}{P}_{v,A}^{I}(t))]+{P}_{i,B}^{R}(t)\delta ,\\ {P}_{i,B}^{I}(t+1) & = & {P}_{i,B}^{I}(t)(1-\gamma )+{P}_{i,B}^{S}(t)\times [1-(1-{p}_{0})\prod _{l\in {\Lambda }_{i,B}}(1-\beta (t){P}_{l,B}^{I}(t))\prod _{v\in {\Lambda }_{i,A}}(1-{\beta }_{ab}{P}_{v,A}^{I}(t))],\\ {P}_{i,B}^{R}(t+1) & = & {P}_{i,B}^{R}(t)(1-\delta )+{P}_{i,B}^{I}(t)\gamma \mathrm{.}\end{matrix}$$

**Figure 6:**
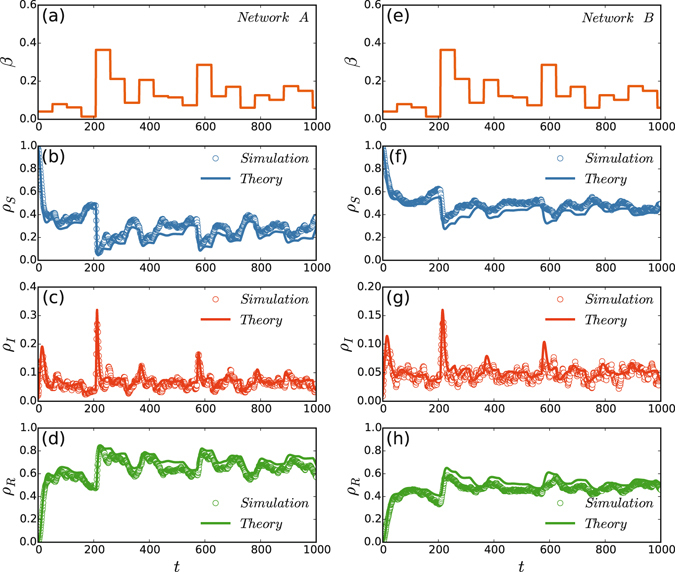
Comparison between the theoretical solutions and numerical simulations. The left and right panels are for the networks ${\mathscr{A}}\,{\rm{and}}\, {\mathcal B}$, respectively. All the parameters are the same as in Fig. 3(a) and (b). (**a**) and (**e**) *β*(*t*) versus *t*; (**b**) and (**f**) *ρ*_*S*_ versus *t*; (**c**) and (**g**) *ρ*_*I*_ versus *t*; (**d**) and (**h**) *ρ*_*R*_ versus *t*. In (**b**–**d**) and (**f**–**h**), the solid curves represent the theoretical solutions while the “circles” represent the numerical simulations.

Instead of getting the analytic solutions of Eqs (6) and (7), we solve them by numerical integration. We set the initial conditions as ${P}_{i,A}^{S}(0)=1.0,{P}_{i,A}^{I}(0)=0.0,{P}_{i,A}^{R}(0)=0.0,{P}_{i,B}^{S}(0)=1.0,{P}_{i,B}^{I}(0)=0.0\,{\rm{and}}\,{P}_{i,B}^{R}(0)=0.0$. To conveniently compare the solutions with the numerical simulations in the section *Results*, we use the same set of *β*(*t*) for both the integration and numerical simulations. Figure 6 shows the results where the left and right panels are for the networks ${\mathscr{A}}$ and ${\mathcal B}$, respectively. In Fig. 6(b–d) and (f–h), the solid curves represent the theoretical solutions while the “circles” represent the numerical simulations. It is easy to see that the theoretical solutions are consistent with the numerical simulations very well.

### A theoretical analysis based on mean field theory

To go deeper into the mechanism of the synchronized and mixed outbreak patterns, we try another theoretical analysis on mean field equations. Let *s* ^*A*^(*t*), *i* ^*A*^(*t*) and *r* ^*A*^(*t*) represent the densities of susceptible, infected, and refractory individuals at time *t* in network ${\mathscr{A}}$, respectively. Similarly, we have *s* ^*B*^(*t*), *i* ^*B*^(*t*) and *r* ^*B*^(*t*) in network ${\mathcal B}$. Then, they satisfy

8 $${s}^{A}(t)+{i}^{A}(t)+{r}^{A}(t)=1,\,{s}^{B}(t)+{i}^{B}(t)+{r}^{B}(t)=1.$$

According to the mean-field theory, we have the following ordinary differential equations

9 $$\frac{d{s}^{A}(t)}{dt}=-\,{p}_{0}{s}^{A}(t)-\beta (t)\langle {k}_{a}\rangle {s}^{A}(t){i}^{A}(t)-{\beta }_{ab}\langle {k}_{ab}\rangle {s}^{A}(t){i}^{B}(t)+\delta {r}^{A}(t),$$

10 $$\frac{d{i}^{A}(t)}{dt}={p}_{0}{s}^{A}(t)+\beta (t)\langle {k}_{a}\rangle {s}^{A}(t){i}^{A}(t)+{\beta }_{ab}\langle {k}_{ab}\rangle {s}^{A}(t){i}^{B}(t)-\gamma {i}^{A}(t),$$

11 $$\frac{d{r}^{A}(t)}{dt}=\gamma {i}^{A}(t)-\delta {r}^{A}(t),$$

12 $$\frac{d{s}^{B}(t)}{dt}=-\,{p}_{0}{s}^{B}(t)-\beta (t)\langle {k}_{b}\rangle {s}^{B}(t){i}^{B}(t)-{\beta }_{ab}\langle {k}_{ab}\rangle {s}^{B}(t){i}^{A}(t)+\delta {r}^{B}(t),$$

13 $$\frac{d{i}^{B}(t)}{dt}={p}_{0}{s}^{B}(t)+\beta (t)\langle {k}_{b}\rangle {s}^{B}(t){i}^{B}(t)+{\beta }_{ab}\langle {k}_{ab}\rangle {s}^{B}(t){i}^{A}(t)-\gamma {i}^{B}(t),$$

14 $$\frac{d{r}^{B}(t)}{dt}=\gamma {i}^{B}(t)-\delta {r}^{B}(t\mathrm{).}$$

Specifically, we consider a case of single epidemic outbreak with extremely weak coupling, i.e. *p*_0_ = 0 and *β*_*ab*_ ≈ 0. In the steady state, we have

15 $$\frac{d{s}^{A}(t)}{dt}=\mathrm{0,}\,\frac{d{i}^{A}(t)}{dt}=\mathrm{0,}\,\frac{d{s}^{B}(t)}{dt}=\mathrm{0,}\,\frac{d{i}^{B}(t)}{dt}=\mathrm{0,}$$

which gives

16 $${s}^{A}(t)=\frac{\gamma }{\beta (t)\langle {k}_{a}\rangle },\,{r}^{A}(t)=\frac{\gamma }{\delta }{i}^{A}(t),\,{s}^{B}(t)=\frac{\gamma }{\beta (t)\langle {k}_{b}\rangle },\,{r}^{B}(t)=\frac{\gamma }{\delta }{i}^{B}(t\mathrm{).}$$

Substituting Eq. (16) into Eq. (8) we obtain

17 $$\frac{\gamma }{\beta (t)\langle {k}_{a}\rangle }+(\frac{\gamma }{\delta }+1){i}^{A}(t)=1,\,\frac{\gamma }{\beta (t)\langle {k}_{b}\rangle }+(\frac{\gamma }{\delta }+1){i}^{B}(t)=1.$$

The critical point can be given by letting *i* ^*A*^(*t*) and *i* ^*B*^(*t*) in Eq. (17) change from zero to nonzero, which gives

18 $${\beta }_{c}^{A}=\frac{\gamma }{\langle {k}_{a}\rangle },\,{\beta }_{c}^{B}=\frac{\gamma }{\langle {k}_{b}\rangle }.$$

For the considered networks with 〈*k*_*a*_〉 > 〈*k*_*b*_〉, we have ${\beta }_{c}^{A} < {\beta }_{c}^{B}$.

### Cross-correlation measure

In statistics, the Pearson correlation coefficient is a measure of the linear correlation between two variables. If two time series {*X*_*t*_} and {*Y*_*t*_} have the mean values $\overline{X}\,{\rm{and}}\,\overline{Y}$, we can define the correlation coefficient *r* as follows

19 $$r=\frac{{\sum }_{t=1}^{n}({X}_{t}-\overline{X})({Y}_{t}-\overline{Y})}{\sqrt{{\sum }_{t=1}^{n}{({X}_{t}-\overline{X})}^{2}\cdot {\sum }_{t=1}^{n}{({Y}_{t}-\overline{Y})}^{2}}}$$

To analyze the correlations of the growth trends between the two time series, we investigates their cross-correlation *r*(*t*) in a given window *w*_*t*_^69, 70^, i.e. $\{{X}_{t},{X}_{t+1},\ldots ,{X}_{t+{w}_{t}}\}\,{\rm{and}}\,\{{Y}_{t},{Y}_{t+1},\ldots ,{Y}_{t+{w}_{t}}\}$. {*X*_*t*_} and {*Y*_*t*_} will share the same trend in the time interval *w*_*t*_ when *r*(*t*) > 0 and the opposite growth trend when *r*(*t*) < 0. In this work, we let the whole time series be one window, i.e. with *w*_*t*_ being the total evolutionary time *t*.

## Electronic supplementary material

Supplementary Information
