# Supplementary material for: Synchronized and mixed outbreaks of coupled recurrent epidemics
Source: Sci Rep. 2017 May 25;7:2424. doi: 10.1038/s41598-017-02661-9 (PMC5445088; doi:10.1038/s41598-017-02661-9)
Supplement: Supplementary file 1 — Supplementary Information [file 41598_2017_2661_MOESM1_ESM.pdf]

Synchronized and mixed outbreaks of coupled recurrent epidemics

Muhua Zheng, Ming Zhao, Byungjoon Min, and Zonghua Liu

I. SUPPLEMENTAL FIGURES FOR THE CASE OF THE ERDŐS-RÉNYI (ER) RANDOM NETWORKS

Fig. 1 shows three more examples of the synchronized and mixed outbreak phases where the coupled networks are based on states-level influenza data and cities-level measles data, respectively.

Fig. 2 shows the distribution of the infected density ρ_I. We see that the distribution is similar to the normal distribution, implying that we can use the normal distribution as the background oscillations of the outbreaks of epidemics.

Fig. 3 shows the evolution of the infected densities ρ_I for the case of $\langle k_a \rangle = \langle k_b \rangle$, confirming the result of $\Delta n = 0$ in main text.

Fig. 4 shows the evolution of the infected densities ρ_I for different inter-layer infectious rate β_{ab}. Fig. 4(a) represents the case of weak coupling where the synchronized and mixed outbreak phases occur in both networks $\mathcal{A}$ and $\mathcal{B}$. Fig. 4(b) represents the case of strong coupling where the mixed outbreak phase is difficult to be observed. Fig. 4(c) represents the case of $\beta_{ab} = \beta(t)$ where the synchronized and mixed outbreak phases can be still observed in the two-layered network.

II. SUPPLEMENTAL FIGURES FOR THE CASE OF SCALE-FREE NETWORKS

Figs. 5-7 show the case of scale-free networks. Fig. 5 corresponds to Fig. 3 and we can't observe the mixed outbreak phase either. Fig. 6 corresponds to Fig. 4 and has the same effect as in Fig.4. Fig. 7 corresponds to Fig. 6 in main text and has the same effect.

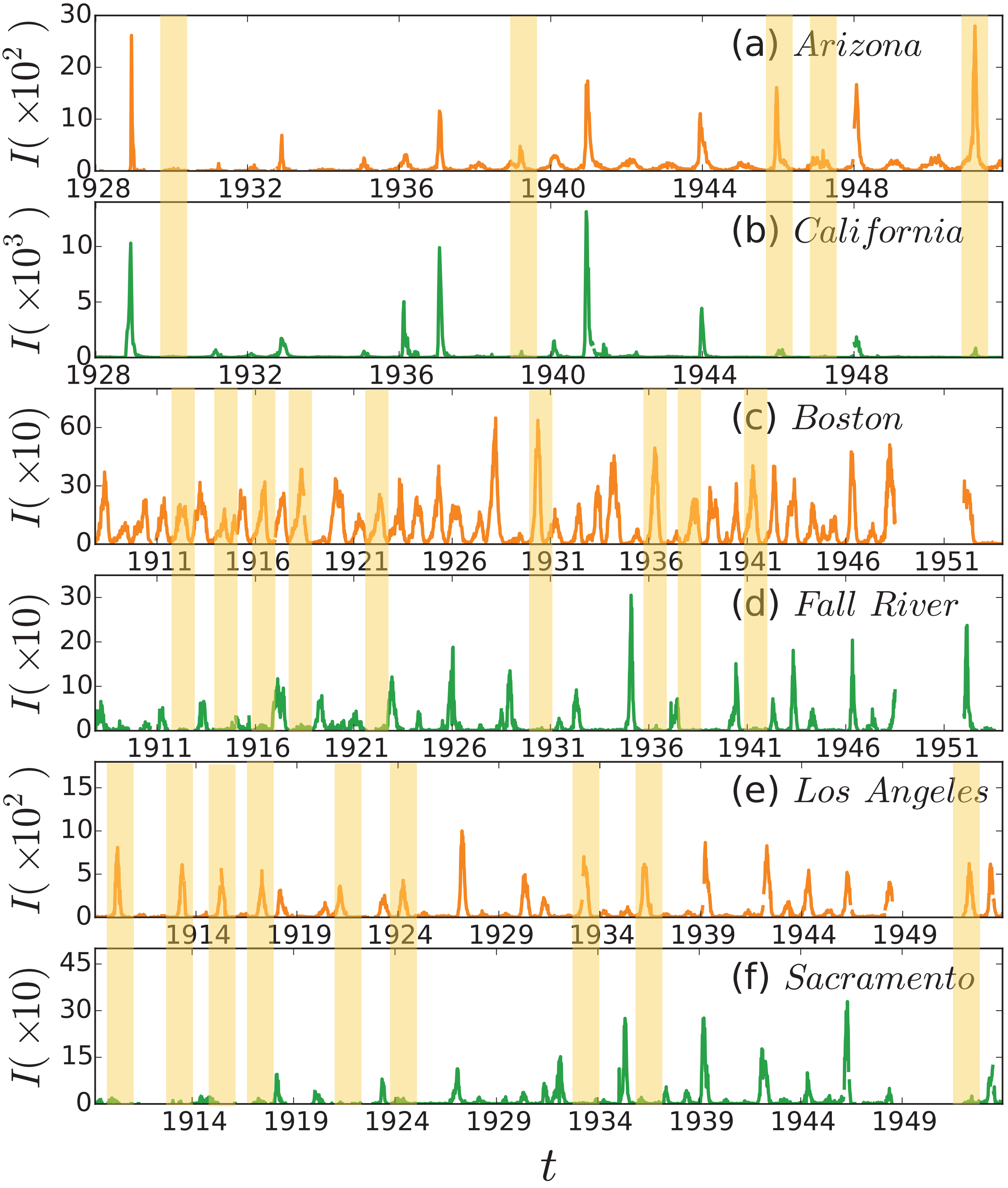

FIG. 1: (color online). **Time series of recurrent epidemics in two coupled states or cities.** (a) and (b) represent the time series of reported weekly influenza infective cases I in the states of Arizona and California, respectively. (c) and (d) represent the time series of reported weekly measles infective cases I in the cities of Boston and Fall River, respectively. (e) and (f) represent the time series of reported weekly measles infective cases I in the cities of Los Angeles and Sacramento, respectively.

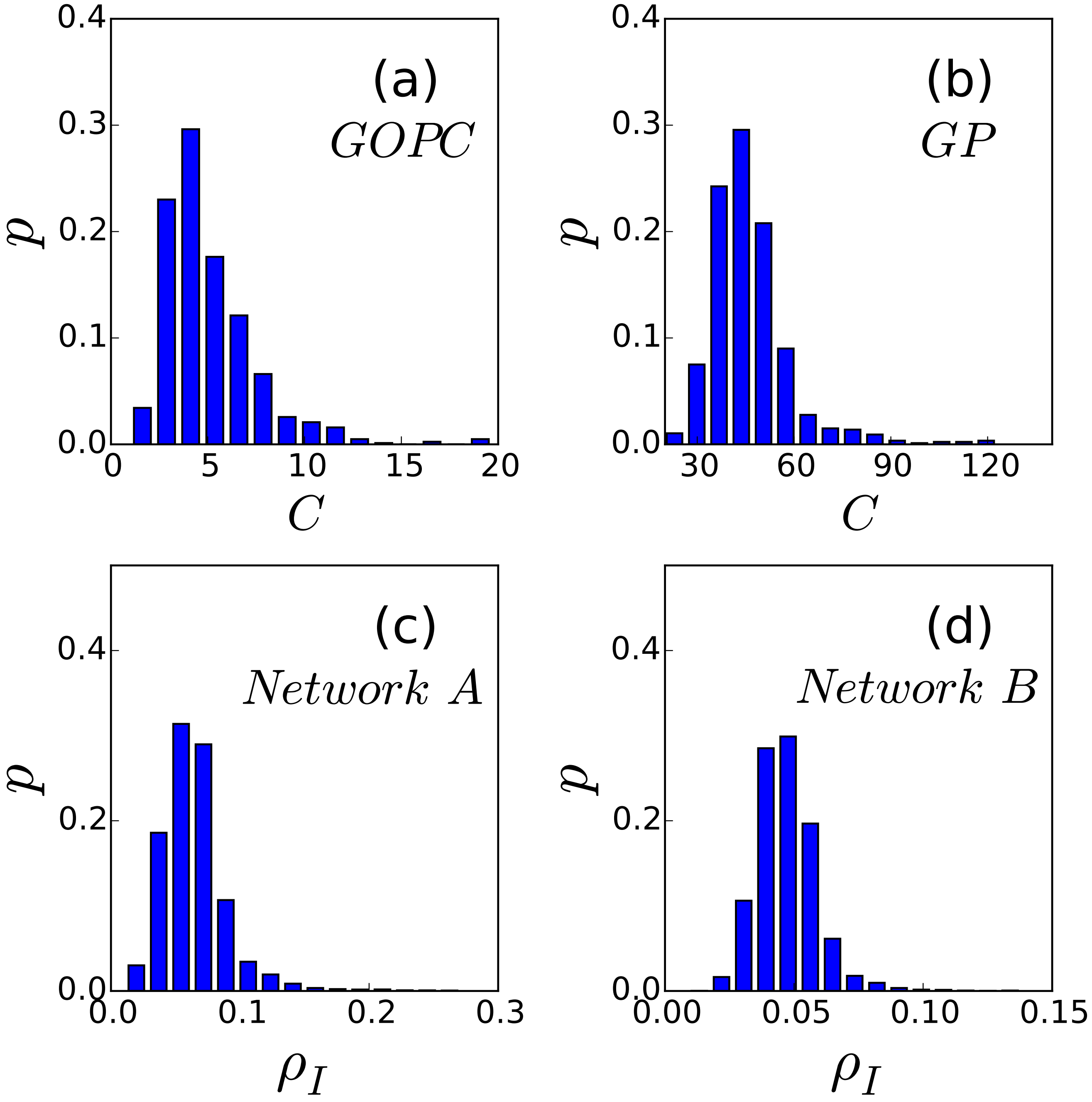

FIG. 2: (color online). **Distribution of the infected density ρ_I.** (a) and (b) represent the cases of the weekly consultation rates of influenza-like illness from the General Out-Patient Clinics (GOPC) and the General Practitioners (GP), respectively. (c) and (d) represent the case of the simulations in Fig. 3 of main text for the networks $\mathcal{A}$ and $\mathcal{B}$, respectively.

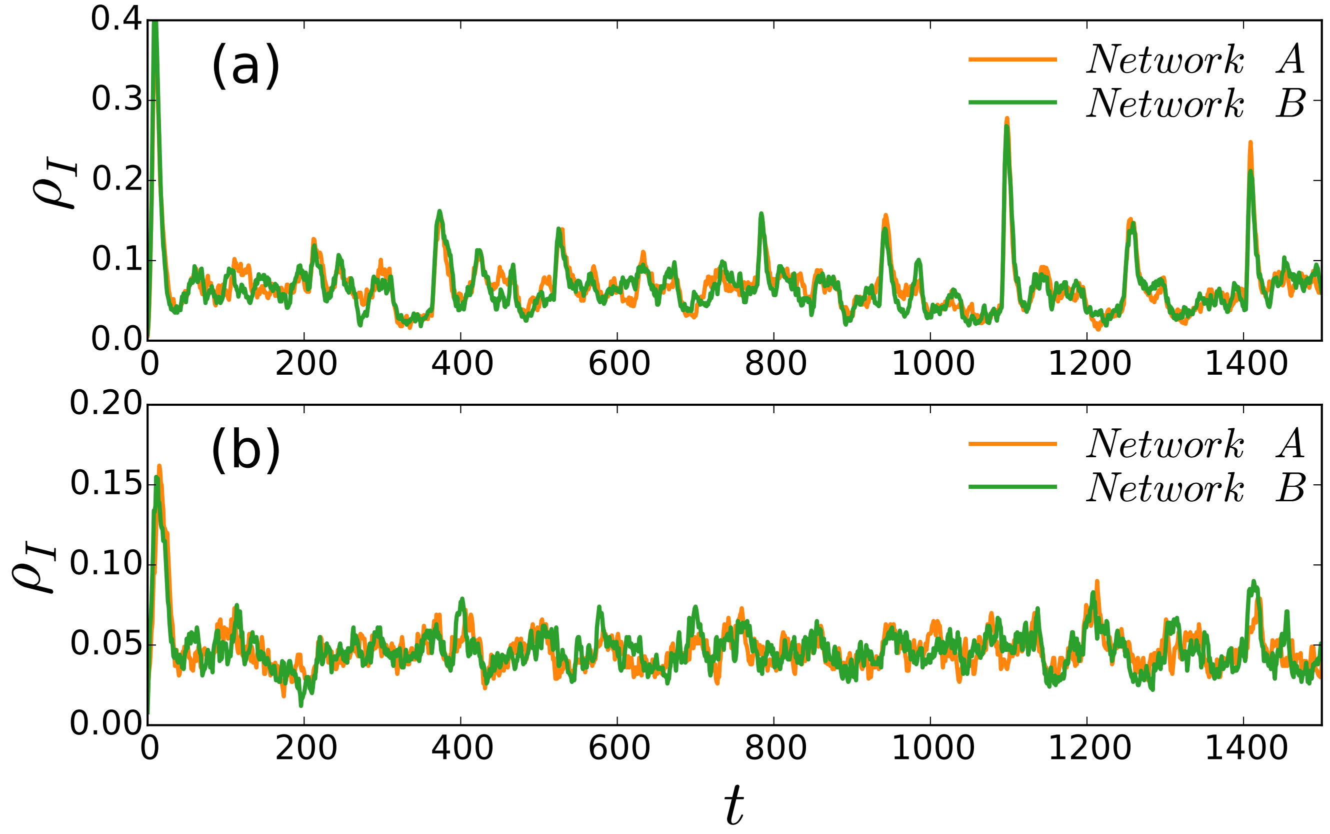

FIG. 3: (color online.) **Evolution of the infected densities ρ_I for the case of $\langle k_a \rangle = \langle k_b \rangle$.** (a) and (b) represent the cases of $\langle k_a \rangle = \langle k_b \rangle = 6.5$ and $\langle k_a \rangle = \langle k_b \rangle = 1.5$, respectively. The other parameters are the same as in Fig. 3 (a) and (b) of main text.

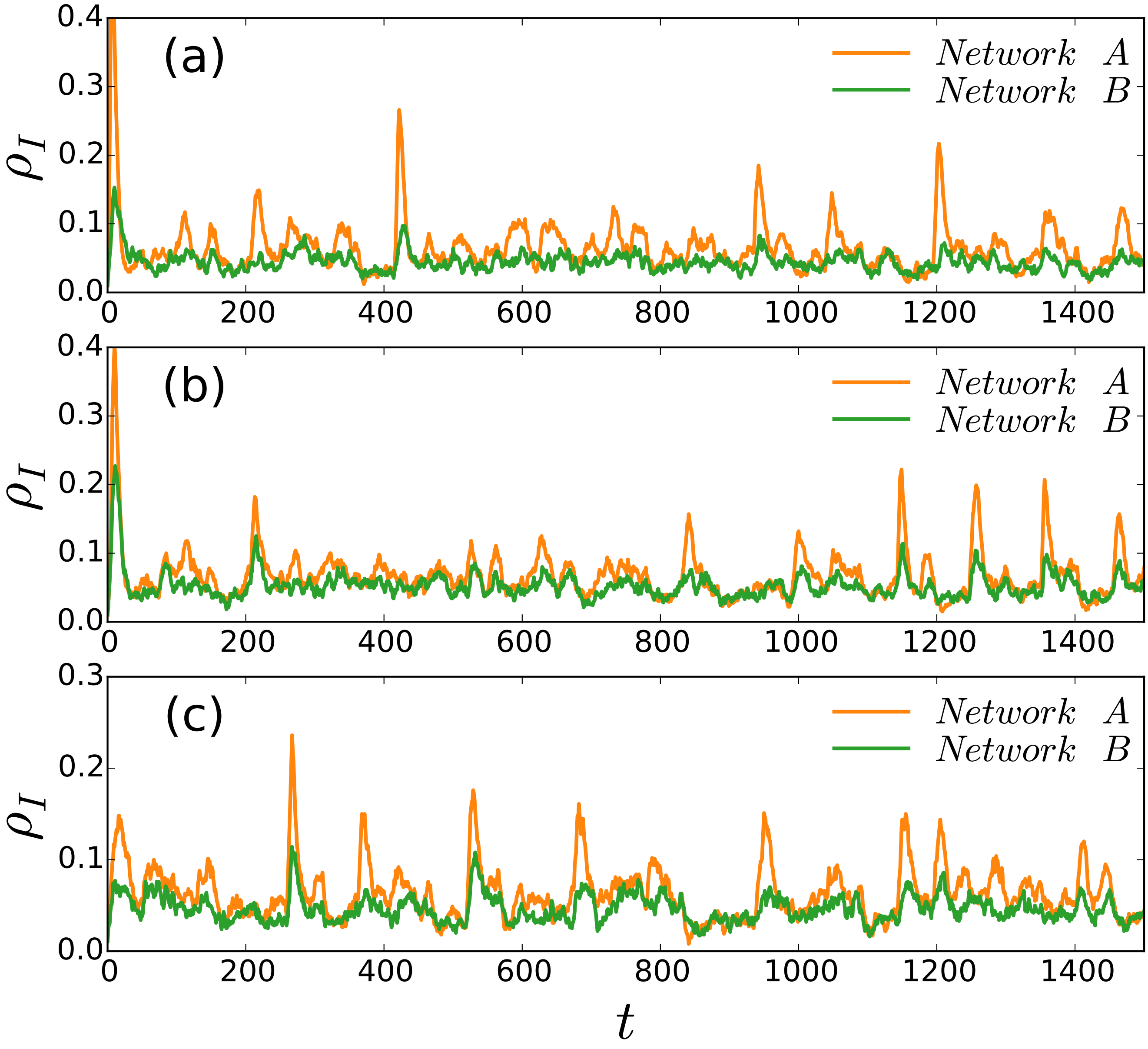

FIG. 4: (color online.) **Evolution of the infected densities ρ_I for different inter-layer infectious rate β_{ab}.** (a)-(c) represent the cases of $\beta_{ab} = 0.02, 0.30$, and the time-dependent coupling $\beta_{ab} = \beta_t$, respectively. The other parameters are the same as in Fig. 3 (a) and (b) of main text.

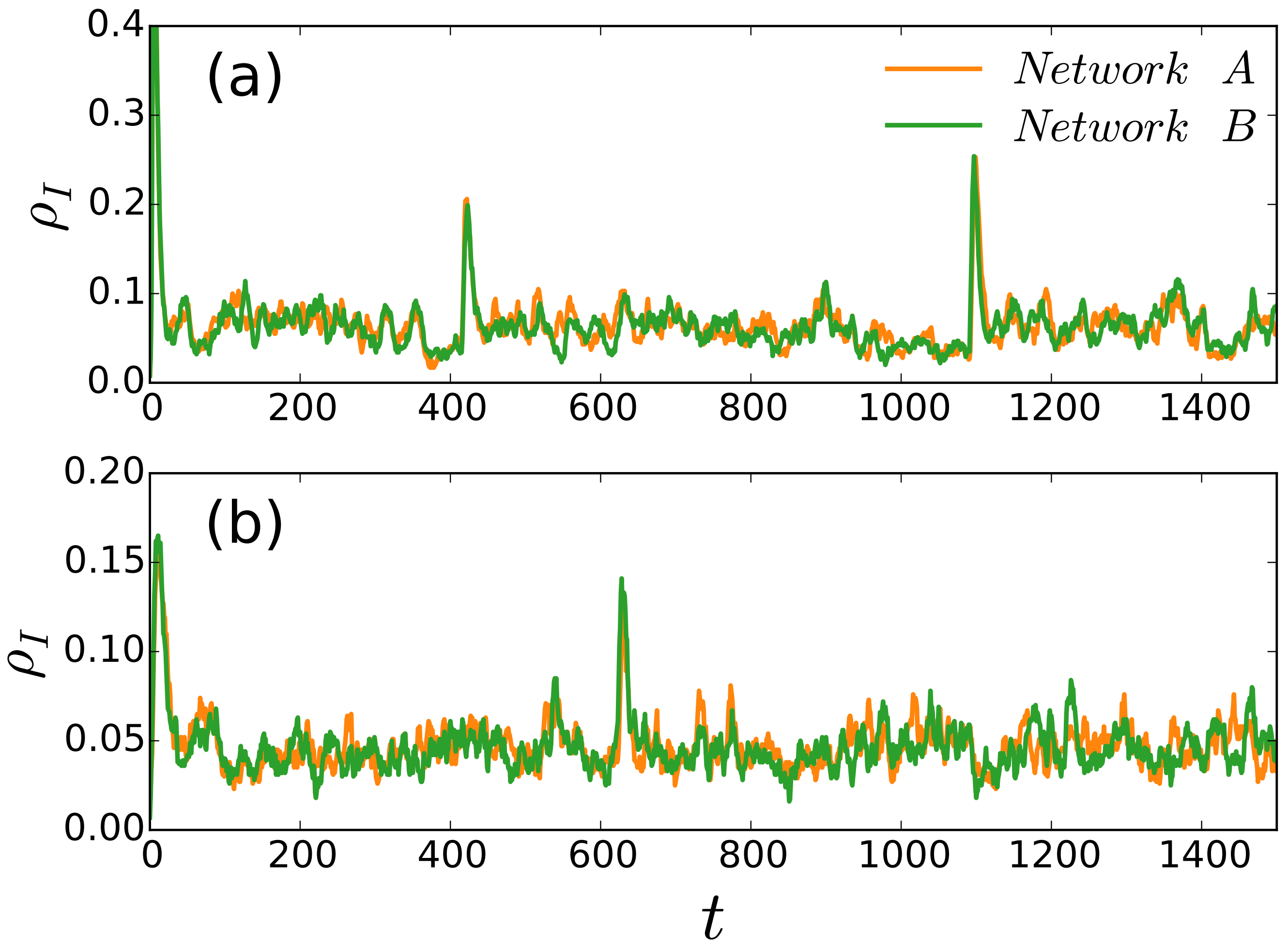

FIG. 5: (color online.) **Evolution of the infected densities ρ_I for the case of $\langle k_a \rangle = \langle k_b \rangle$ in scale-free networks.** (a) and (b) represent the cases of $\langle k_a \rangle = \langle k_b \rangle = 6.5$ and $\langle k_a \rangle = \langle k_b \rangle = 1.5$, respectively, which corresponds to Fig. 3.

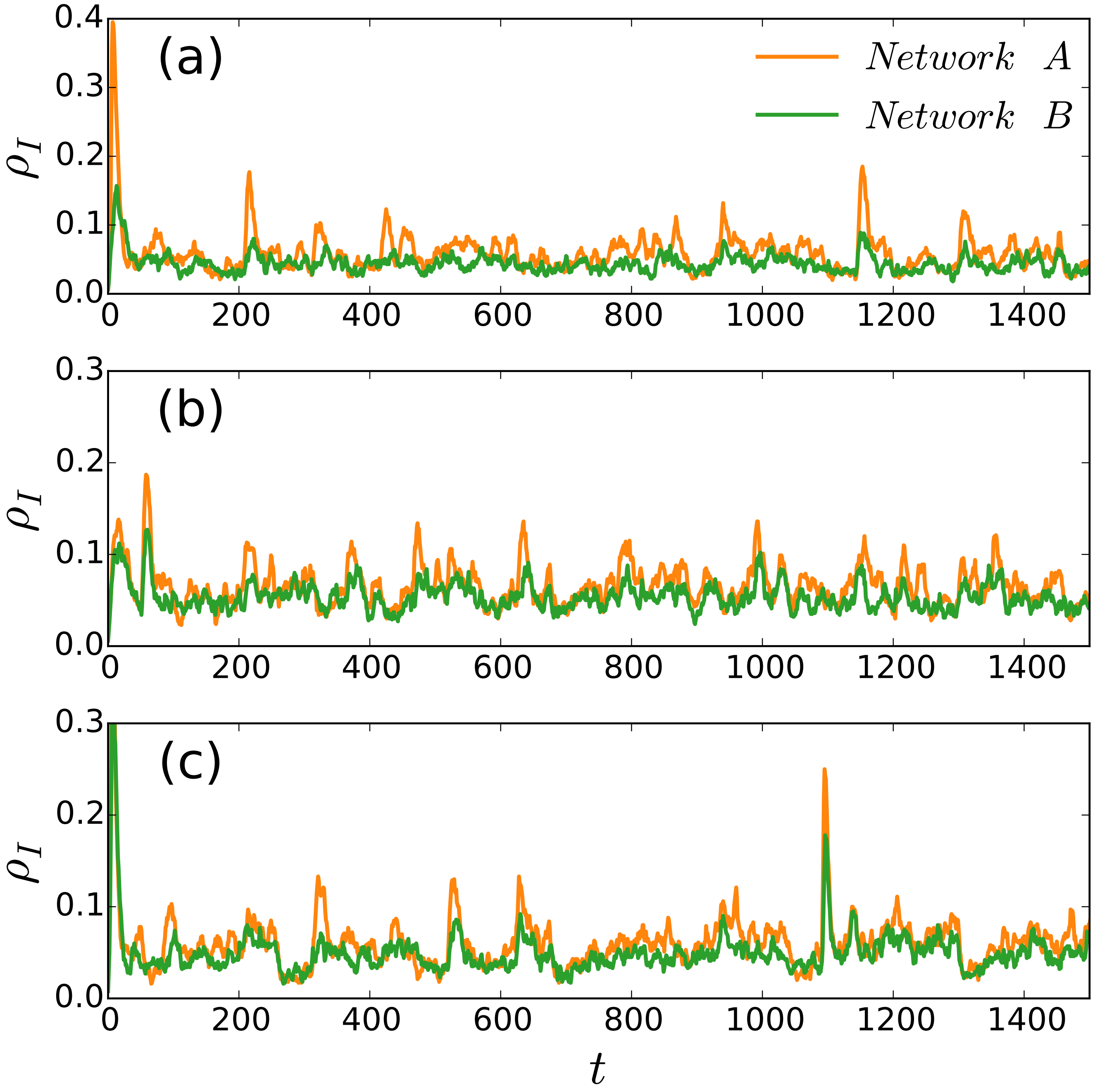

FIG. 6: (color online.) **Evolution of the infected densities ρ_I for different inter-layer infectious rate β_{ab} in scale-free networks.** (a)-(c) represent the cases of $\beta_{ab} = 0.02, 0.30$, and the time-dependent coupling $\beta_{ab} = \beta_t$, respectively, which corresponds to Fig. 4.

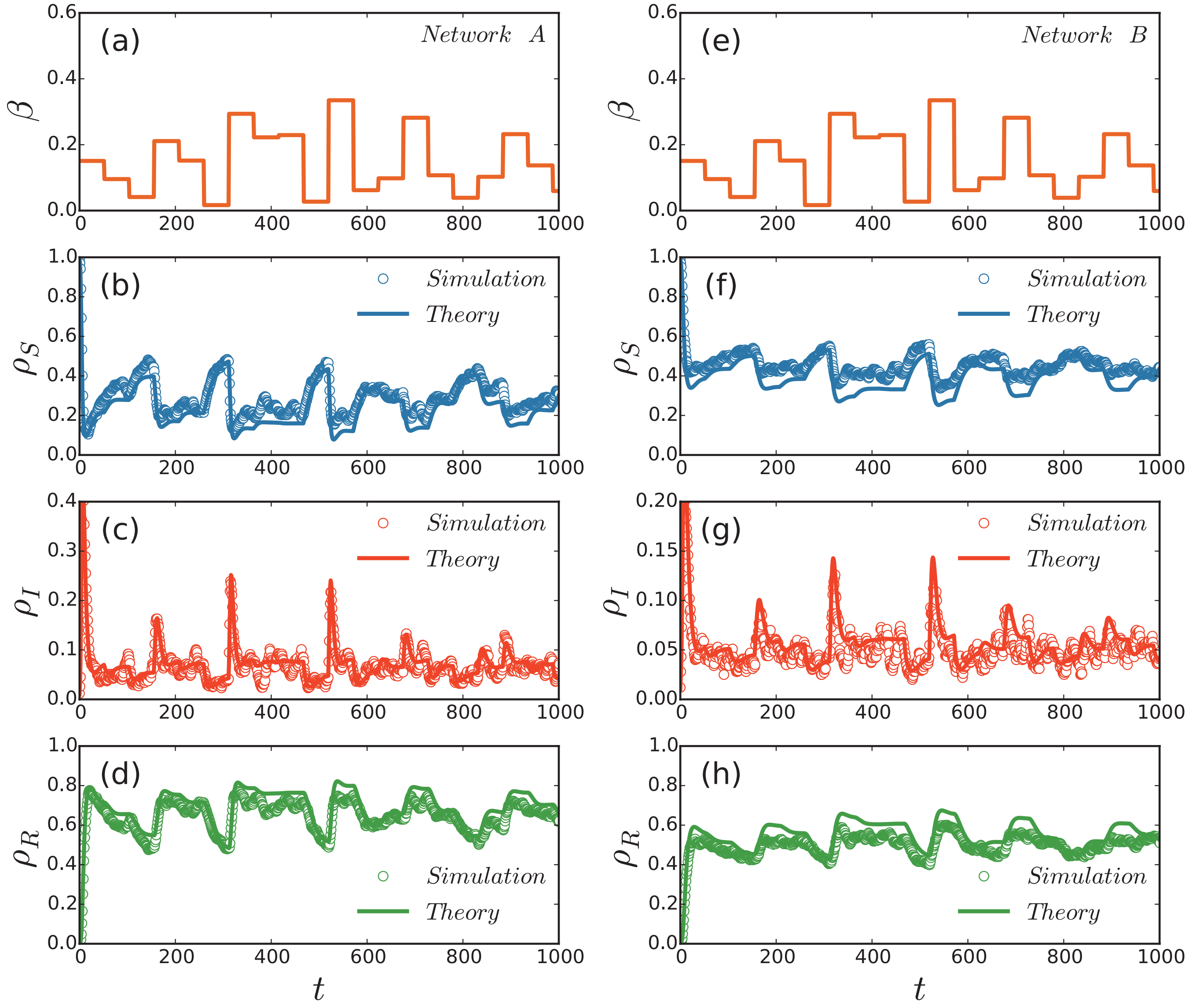

FIG. 7: (color online.) **Comparison between the theoretical solutions and numerical simulations in scale-free networks.** The left and right panels are for the networks $\mathcal{A}$ and $\mathcal{B}$, respectively. (a) and (e) $\beta(t)$ versus t; (b) and (f) ρ_S versus t; (c) and (g) ρ_I versus t; (d) and (h) ρ_R versus t. In (b)-(d) and (f)-(h), the solid curves represent the theoretical solutions while the "circles" represent the numerical simulations. This figure corresponds to Fig. 6 in main text.
